# Targeting PIN1 as a Therapeutic Approach for Hepatocellular Carcinoma

**DOI:** 10.3389/fcell.2019.00369

**Published:** 2020-01-15

**Authors:** Chi-Wai Cheng, Eric Tse

**Affiliations:** Department of Medicine, The University of Hong Kong, Pokfulam, Hong Kong

**Keywords:** PIN1, phosphorylation, hepatocellular carcinoma, inhibitor, hepatocarcinogenesis

## Abstract

PIN1 is a peptidyl-prolyl *cis/trans* isomerase that specifically binds and catalyzes the *cis/trans* isomerization of the phosphorylated serine or threonine residue preceding a proline (pSer/Thr-Pro) motif of its interacting proteins. Through this phosphorylation-dependent prolyl isomerization, PIN1 is involved in the regulation of various important cellular processes including cell cycle progression, cell proliferation, apoptosis and microRNAs biogenesis; hence its dysregulation contributes to malignant transformation. PIN1 is highly expressed in hepatocellular carcinoma (HCC). By fine-tuning the functions of its interacting proteins such as cyclin D1, x-protein of hepatitis B virus and exportin 5, PIN1 plays an important role in hepatocarcinogenesis. Growing evidence supports that targeting PIN1 is a potential therapeutic approach for HCC by inhibiting cell proliferation, inducing cellular apoptosis, and restoring microRNAs biogenesis. Novel formulation of PIN1 inhibitors that increases *in vivo* bioavailability of PIN1 inhibitors represents a promising future direction for the therapeutic strategy of HCC treatment. In this review, the mechanisms underlying PIN1 over-expression in HCC are explored. Furthermore, we also discuss the roles of PIN1 in HCC tumorigenesis and metastasis through its interaction with various phosphoproteins. Finally, recent progress in the therapeutic options targeting PIN1 for HCC treatment is examined and summarized.

## Introduction

Hepatocellular carcinoma (HCC) is the fifth most common cancer in men and the ninth in women worldwide. It is the third leading cause of cancer death, with a reported mortality of more than 780,000 per year (Bray et al., 2018). Patients with HCC have poor outcome and have an inferior 5-year overall survival of 18% as compared with that of other common cancers including breast (90%), colon (65%), prostate (98%), and stomach (31%) cancers (American_Cancer_Society, 2019). Chronic infection with hepatitis B virus (HBV) or hepatitis C virus (HCV), heavy alcohol consumption and the presence of liver cirrhosis are important risk factors for the development of HCC (Rawla et al., 2018). At the cellular level, metabolic dysregulation and genetic aberrations contribute to HCC development through the activation of multiple cancer-driving signaling pathways. The interplay of these signaling pathways results in a complex cancer cell circuitry that leads to the aggressive clinical course and poor treatment outcomes of HCC. Early stage HCCs are amenable to several curative and effective therapies including orthotopic liver transplantation, surgical liver resection, transcatheter arterial chemoembolization (TACE) and radiofrequency ablation (RFA) (Lurje et al., 2019). However, only a small proportion of HCC cases are detected at an early stage, owing to the lack of sensitivity of the conventional HCC surveillance techniques such as ultrasonography (60%) and computed tomography (68%) (Colli et al., 2006). As a result, HCCs are frequently diagnosed at advanced stage and curative treatment options are not available for these patients. Conventional chemotherapy and molecular targeting therapy for advanced HCC have modest efficacy only. Clinical studies have demonstrated that treatment with doxorubicin or tyrosine kinase inhibitor sorafenib extend the survival of patients with advanced HCC for merely 3 and 12 weeks, respectively (Lai et al., 1988; Llovet et al., 2008). These treatments are also associated with development of drug resistance and ultimate disease progression (Chow et al., 2013; Nishida et al., 2015; Zheng, 2017). Recently, several molecular targeting drugs, including lenvatinib, regorafenib, and cabozantinib, have been approved by the US Food and Drug Administration (FDA) for the treatment of advanced HCC. Similar to sorafenib, lenvatinib is recommended as a first-line therapeutic agent for patients with advanced HCC. The other two molecular targeting drugs are approved as a second-line treatment in the presence of sorafenib resistance. Lenvatinib, regorafenib and cabozantinib are tyrosine kinase inhibitors and through inhibiting different sets of tyrosine kinases, treatment with these inhibitors results in better overall survival benefit as compared with sorafenib (Table 1; Bruix et al., 2017; El-Khoueiry et al., 2017; Abou-Alfa et al., 2018; Kudo et al., 2018). Thus, it is imperative to understand the various signaling pathways involved in hepatocarcinogenesis to facilitate the development of effective molecular targeting drugs.

**TABLE 1:** Molecular targeting drugs for hepatocellular carcinoma.

**Drug**	**Targets**	**Study phase**	**Overall survival**	**References**
**First-line treatment**				
Sorafenib	VEGFR1/2/3; PDGFR; RAF/MEK/ERK	III	10.7 months	Llovet et al., 2008; Cheng et al., 2009
		III	6.5 months	
Lenvatinib	VEGFR1/2/3; FGFR1/2/3/4; FGF; PDGFR; RET	III	13.6 months	Kudo et al., 2018
**Second-line treatment**				
Regorafenib	VEGFR1; RET; RAF1; TIE2; BRAF; PDGFR; FGFR	II	13.8 months	Bruix et al., 2017
Cabozantinib	VEGFR1/2/3; c-MET	III	10.2 months	Abou-Alfa et al., 2018
Nivolumab	PD-1	I/II	15 months	El-Khoueiry et al., 2017

*VEGFR, Vascular endothelial growth factor receptor; PDGFR, Platelet-derived growth factor receptor; FGFR, Fibroblast growth factor receptor; RET, Glial cell-derived neurotrophic factor receptor; TIE2, Angiopoietin receptor; c-MET, Hepatocyte growth factor receptor; PD-1, Programmed cell death protein 1.*

Cancer-driving signaling pathways are often regulated by protein phosphorylation and dephosphorylation. Phosphorylation of serine or threonine residues preceding proline (pSer/Thr-Pro) motif of many regulatory proteins is mediated by cyclin-dependent kinases (CDKs) and mitogen-activated protein kinases (MAPKs). The phosphorylated Ser/Thr-Pro motif provides a potential binding site for the peptidyl-prolyl *cis/trans* isomerase PIN1 that catalyzes a *cis/trans* isomerization of the prolyl peptide bond (Lu et al., 1996; Lu, 2000). PIN1 is mainly localized in the nucleus and consists of two structurally and functionally distinct domains (Lee et al., 2011). Its N-terminal WW domain is responsible for specific binding to the pSer/Thr-Pro motifs of its protein substrates while its C-terminal prolyl isomerase (PPIase) domain is responsible for catalyzing *cis/trans* isomerization of the pSer/Thr-Pro peptide bonds (Lu et al., 1999; Lu P. J. et al., 2002; Behrsin et al., 2007). PIN1-mediated isomerization induces conformational changes of its bound proteins, thereby fine-tuning their cellular functions, interactions with other proteins, stability and subcellular localization (Lu K. P. et al., 2002). Through this mechanism, PIN1 is involved in various cellular processes, including apoptosis, cell cycle progression, cell proliferation, differentiation and transformation. As a result, PIN1 plays an important role in many human diseases including Alzheimer’s disease (AD) and cancers (Zhou and Lu, 2016).

In cancer, PIN1 has been shown to promote carcinogenesis through its interaction with cell-cycle regulatory proteins and apoptosis-related proteins including β-catenin, cyclin D1, nuclear factor-kappa B (NF-κB)-p65, p53, and myeloid cell leukemia-1 (Mcl-1) (Ryo et al., 2001; Liou et al., 2002; Zacchi et al., 2002; Ryo et al., 2003; Ding et al., 2008). These PIN1-interacting proteins are frequently deregulated in cancers, and their oncogenic potential is enhanced through PIN1-dependent isomerization. Consequently, PIN1 over-expression has been linked to dysregulated cell proliferation, malignant transformation and tumor development. Indeed, PIN1 over-expression has been found in many cancers, including hepatocellular carcinoma (HCC). Several studies have shown that PIN1 is over-expressed in more than 50% of HCC tissues (Pang et al., 2004; Cheng et al., 2013; Shinoda et al., 2015; Leong et al., 2017). In addition, PIN1 over-expression not only promotes malignant transformation of hepatocytes (Pang et al., 2006), but also enhances hepatocarcinogenesis through interaction with the x-protein of hepatitis B virus (HBx), the inhibitor of apoptosis protein survivin, and the cycle-dependent kinase inhibitor p27 (Pang et al., 2007; Cheng et al., 2013, 2017). Notably, compelling evidence shows that inhibition of PIN1 suppresses the proliferation of HCC cells *in vitro* and *in vivo* (Liao et al., 2017; Zheng et al., 2017; Pu et al., 2018; Yang et al., 2018; Sun et al., 2019). Currently, there is no effective conventional chemotherapy and molecular targeting therapy for advanced HCC. Thus, PIN1 inhibition may be a promising therapeutic strategy for HCC treatment. In this article, we review the role of PIN1 in HCC and discuss the therapeutic potential of targeting PIN1.

## Regulation of Pin1 Expression in Hepatocellular Carcinoma

Many studies have demonstrated a high prevalence of PIN1 over-expression in HCC. The expression of PIN1 is regulated by a number of transcriptional factors and microRNAs (miRNAs). miRNAs are a family of small non-coding RNAs that negatively regulate gene expression by binding to the 3′UTR of target mRNA, resulting in the target mRNA degradation or translational repression. Currently, six miRNAs (miR-140-5p, miR-200b/c, miR-296-5p, miR-370, and miR-874-3p) (Table 2) have been found to bind PIN1 mRNA directly and inhibit its expression in cancers (Zhang et al., 2013; Lee et al., 2014; Luo et al., 2014; Leong et al., 2017; Yan et al., 2017; Chen et al., 2018). Experiments have confirmed that over-expression of these miRNAs reduces PIN1 protein expression in cancer cells and reverses PIN1-mediated cellular effects, including cell proliferation, apoptosis, migration and invasion. Among these PIN1-targeting miRNAs, the expression of miR-140-5p and miR-874-3p are significantly down-regulated and inversely correlated with PIN1 overexpression in primary human HCC samples, suggesting that the down-regulation of miR-140-5p and miR-874-3p contributes to PIN1 over-expression during hepatocarcinogenesis.

**TABLE 2:** Identification of PIN1-targeting microRNAs.

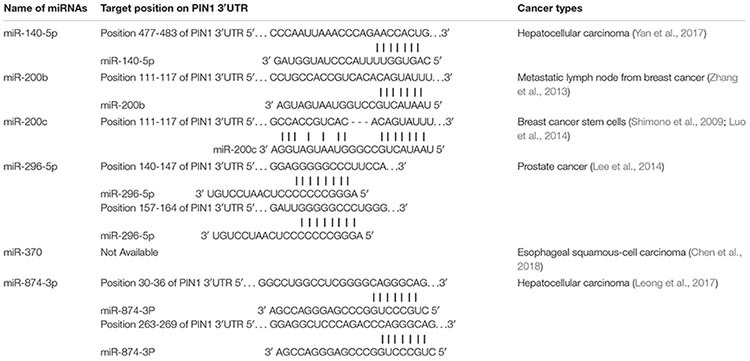

*miRNA, microRNA; UTR, Untranslated region.*

PIN1 expression is also transcriptionally regulated by retinoblastoma protein (Rb)-E2F pathway. E2F protein is a transcription factor that activates PIN1 expression by binding to the E2F-binding sites of the *PIN1* gene promoter (Ryo et al., 2002). Hypophosphorylated Rb binds to and sequesters E2F transcription factor, leading to transcriptional inactivation of PIN1 expression. After phosphorylation by CDK kinases, hyperphosphorylated Rb dissociates E2F transcription factors from Rb-E2F complex, resulting in increased E2F transcriptional activity and PIN1 expression. Therefore, the E2F-induced PIN1 expression mainly depends on the release of E2F transcription factor from the hyperphosphorylated Rb. As a higher nuclear expression of E2F protein is found in HCC tissues (Palaiologou et al., 2012), it is speculated that a higher E2F expression may contribute to PIN1 over-expression in HCC pathogenesis.

The relationships between PIN1 expression and clinical factors in HCC have also been studied (Shinoda et al., 2015). Higher PIN1 expression is significantly associated with larger tumor size, increased intrahepatic metastasis and portal vein invasion. Compared with patients with low PIN1 expression, patients with high PIN1 expression show significantly inferior prognosis, shorter overall survival and higher early recurrence rate. These findings support the notion that deregulated PIN1 expression may play an important role in determining the clinical course of HCC.

## Roles of Pin1 in Hepatocarcinogenesis

**FIGURE 1:**
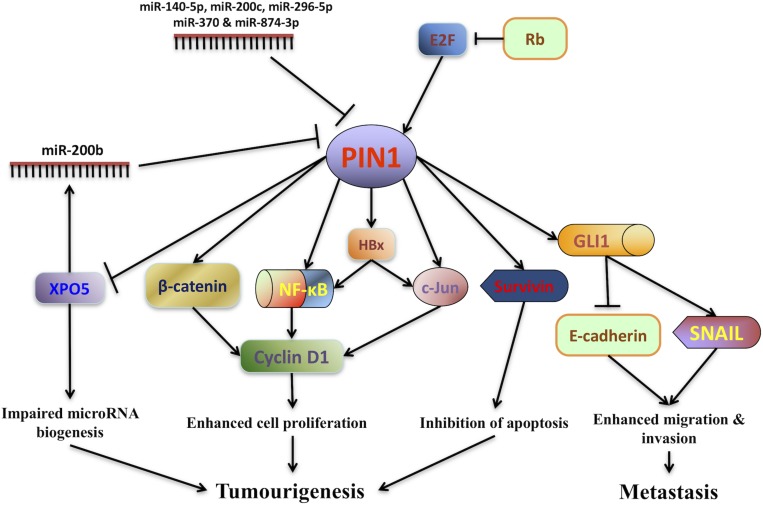
PIN1 involvement in hepatocarcinogenesis. The expression of PIN1 is up-regulated by E2F transcription factors through the retinoblastoma protein (Rb)-E2F pathway while its expression is down-regulated by a number of microRNAs (miR-140-5p, miR-200b/c, miR-296-5p, miR-370, and miR-874-3p). Through phosphorylation-dependent prolyl isomerization, PIN1 fine-tunes the oncogenic functions of various phosphoproteins involved in HCC tumorigenesis. PIN1 increases cyclin D1 protein level through both transcriptional and translational regulation. At the transcriptional level, PIN1 increases the transcriptional activities of β-catenin, c-Jun and nuclear factor-kappa B (NF-κB), resulting in an increase in cyclin D1 transcription. At the translational level, PIN1 binds and stabilizes cyclin D1 protein. In addition, PIN1 stabilizes Hepatitis B virus X-protein (HBx) and augments HBx transactivating activity on downstream targets c-Jun and NF-κB that up-regulate cyclin D1 expression. Increased cyclin D1 expression in turn contributes to enhanced cell proliferation and tumorigenesis. PIN1 also enhances the anti-apoptotic function of survivin to inhibit caspase-dependent apoptosis and promote tumorigenesis. Furthermore, PIN1 impairs pre-miRNA binding capacity of exportin-5 (XPO5), decreases nuclear to cytoplasmic export of pre-miRNA and finally reduces miRNA biogenesis. Global down-regulation of miRNA expression leads to HCC tumorigenesis. Through interaction with GLI1, PIN1 enhances migration and invasion of HCC cells by up-regulating mesenchymal cell marker SNAIL and down-regulating epithelial cell marker E-cadherin.

The first evidence showing the role of PIN1 in promoting HCC pathogenesis is the finding of malignant transformation of non-tumorigenic human liver cells by PIN1 over-expression (Pang et al., 2006). Both *in vitro and in vivo* experiments have demonstrated that PIN1 over-expression in non-tumorigenic liver cells induces the colony formation in soft agar and tumor formation in nude mice. Conversely, PIN1 depletion by shRNA reduces HCC tumorigenicity (Pang et al., 2006; Cheng et al., 2013). Through PIN1-mediated isomerization, PIN1 contributes to hepatocarcinogenesis by fine-tuning the oncogenic functions of its interacting proteins (Figure 1).

## Pin1 and Cyclin D1

One of the most well studied oncogenic proteins regulated by PIN1 is cyclin D1, an important cell cycle regulator. Deregulation of cyclin D1 is associated with various types of cancers. Cyclin D1 functions to trigger the cell entering cell cycle and its expression is critical for promoting cell cycle progression and cell proliferation (Hunter and Pines, 1994). Notably, PIN1 has been found to interact with cyclin D1 in cancer cells. Through PIN1-dependent isomerization, PIN1 increases the protein stability of cyclin D1, resulting in the increase of nuclear accumulation of cyclin D1 (Liou et al., 2002). In addition to its post-translational regulation, PIN1 also increases cyclin D1 expression at the transcriptional level. Firstly, PIN1 interacts with β-catenin to inhibit nuclear export and protein degradation of β-catenin (Ryo et al., 2001). Increased nuclear accumulation of β-catenin leads to an increase of β-catenin transcriptional activity on the downstream target genes, including *cyclin D1*. Moreover, PIN1 has been found to bind c-Jun and the p65/RelA subunit of NF-κB, leading to the activation of c-Jun and NF-κB transcriptional activities toward the *cyclin D1* gene (Wulf et al., 2001; Ryo et al., 2003). Consequently, PIN1 over-expression increases cyclin D1 protein expression level through PIN1-mediated protein stabilization of cyclin D1 and PIN1-induced transcriptional activation of β-catenin, c-Jun and NF-κB. Increased cyclin D1 expression in turn promotes cell proliferation. In fact, several studies have demonstrated that PIN1 expression is positively correlated with the cyclin D1 expression in human HCC tumors (Pang et al., 2004; Shinoda et al., 2015), further confirming the role of PIN1 in promoting hepatocarcinogenesis by up-regulation of cyclin D1 expression.

## Pin1 and HBx

Chronic infection with HBV contributes to hepatocarcinogenesis through several mechanisms. These mechanisms involve the integration of HBV-DNA into the host genome to induce chromosome instability, insertional mutagenesis of diverse cancer-related genes to alter their expression, and the expression of viral regulatory protein HBx to modulate apoptosis and cell proliferation of the infected cells (Brechot et al., 1980; Feitelson and Duan, 1997; Paterlini-Brechot et al., 2003). HBx is known to interact with p53 and inhibits its translocation into the nucleus, resulting in the suppression of p53-dependent apoptosis (Ueda et al., 1995). HBx is also a viral transactivator that promotes cell proliferation by up-regulating the expression of several oncogenes including c-Jun and NF-κB. Previously, our group demonstrates an interaction between PIN1 and HBx (Pang et al., 2007). PIN1-dependent isomerization of HBx results in stabilization of the HBx protein and augmentation of the HBx transactivating activity. Enhanced transactivation activity of HBx resulting from PIN1 and HBx interaction up-regulates the expression of its downstream target gene NF-κB. As a result, co-expression of PIN1 and HBx synergistically promotes cell proliferation and xenograft tumor growth in HCC as compared with the expression of PIN1 or HBx alone (Pang et al., 2007). In addition, PIN1 over-expression is strongly associated with HBV-related HCC tumors, suggesting that PIN1 is critical for HBV-induced hepatocarcinogenesis through its upregulation of the transactivating activity of HBx protein.

## Pin1 and Survivin

Deregulation of apoptosis is also involved in HCC pathogenesis. PIN1 has been found to interact with the anti-apoptotic protein survivin in HCC cells (Cheng et al., 2013). During mitotic progression of a proliferating cell, phosphorylation of survivin on Thr^34^-Pro motif occurs to facilitate its binding to hepatitis B X interacting protein (HBXIP) and pro-caspase-9, thereby preventing caspase-9 activation and inhibiting apoptosis (Marusawa et al., 2003). Interestingly, this survivin phosphorylation site (Thr^34^-Pro) is also a binding site for PIN1. Through PIN1-dependent isomerization, PIN1 increases the binding between survivin and pro-caspase-9 via HBXIP, resulting in the suppression of caspase-dependent apoptosis in HCC cells (Cheng et al., 2013). Inhibition of apoptosis by PIN1 results in an increase of tumor growth in HCC xenograft mouse model. In addition, both PIN1 and survivin protein expression levels are higher in human HCC tumors as compared with adjacent non-timorous liver tissues, and there is a positive correlation between PIN1 and survivin expression in HCC (Cheng et al., 2013). These findings suggest that PIN1 over-expression promotes hepatocarcinogenesis by enhancing the anti-apoptotic function of survivin.

## Pin1 and XPO5

As discussed earlier, several miRNAs have been found to inhibit PIN1 expression in cancers. Conversely, PIN1 also regulates miRNAs expression through its interaction with precursor-miRNA (pre-miRNA) transporter exportin-5 (XPO5) (Li et al., 2018). The biogenesis of miRNA starts with the transcription of a primary miRNA (pri-miRNA) by RNA polymerase II, followed by processing of the pri-miRNA to generate pre-miRNA by Drosha in the nucleus. Nuclear pre-miRNA with short hairpin structure only becomes a functional mature miRNA after cytoplasmic processing by Dicer (Krol et al., 2010). The function of XPO5 is to mediate the export of pre-miRNA from nucleus to cytoplasm for miRNA maturation. As pre-miRNA export is a rate-limiting step in miRNA biogenesis, XPO5 plays a critical role in the regulation of miRNA expression (Yi et al., 2005).

In HCC cells, phosphorylation of XPO5 by ERK has been shown to promote its interaction with PIN1 (Sun et al., 2016). Through PIN1-mediated isomerization, PIN1 impairs the pre-miRNA binding capacity of XPO5, resulting in the suppression of nuclear-cytoplasmic export of pre-miRNA (Li et al., 2018). Reduced cytoplasmic pre-miRNA finally reduces miRNA biogenesis and miRNA expression. By impairing pre-miRNA binding capacity of XPO5, PIN1 has been found to reduce the expression of tumor suppressive miRNAs, such as miR-122, miR-200b, and miR-146a. Through decreasing the expression of these miRNAs in HCC cells, PIN1 promotes cell proliferation *in vitro* and tumor growth *in vivo*. In fact, a global down-regulation of miRNA expression is tightly associated with HCC progression (Wong et al., 2012). Given the critical role of nuclear-cytoplasmic export of pre-miRNA in miRNA biogenesis, the regulation of pre-miRNA binding ability of XPO5 by PIN1 has profound effects on miRNA expression, which in turn contributes to hepatocarcinogenesis (Li et al., 2018). Interestingly, PIN1 has been shown to suppress the expression of miR-200b that is one of the PIN1-targeting miRNAs (Zhang et al., 2013; Li et al., 2018). Further study is required to investigate this potential positive feedback loop for PIN1 over-expression through the regulation of miR-200b biogenesis in HCC.

## Pin1 and Gli1

There is increasing evidence to indicate that PIN1 enhances migration and invasion of cancer cells by promoting epithelial-mesenchymal transition (EMT). PIN1 over-expression in breast cancer cells has been found to increase expression of mesenchymal cell markers such as N-cadherin, SNAIL and vimentin, and decrease expression of epithelial cell marker E-cadherin (Kim et al., 2009; Luo et al., 2014). The first evidence showing the role of PIN1 in enhancing migration and invasion ability of HCC cells is the identification of its interaction with GLI1, an effector of the Hedgehog pathway (Wang et al., 2019). GLI1 is a transcriptional factor that activates Hedgehog signals, thereby promoting EMT and HCC metastasis (Li et al., 2016). PIN1 interacts with and stabilizes GLI1 in HCC cells, leading to an increase in GLI1 protein expression. Although the mechanism underlying the regulation of EMT by GLI1 remains undefined, PIN1-induced GLI1 stabilization results in an altered expression of EMT regulating proteins with up-regulation of SNAIL and down-regulation of E-cadherin (Wang et al., 2019). Experiments have confirmed that PIN1 over-expression promotes HCC cell migration and invasion *in vitro* while its depletion by shRNA inhibits lung metastasis of HCC cells *in vivo* (Wang et al., 2019). Interestingly, PIN1 expression is not only positively correlated with GLI1, but also with SNAIL in human HCC tissues. Moreover, PIN1 expression is also negatively correlated with E-cadherin in HCC (Wang et al., 2019). These findings have demonstrated that PIN1 contributes to migration and invasion of HCC cells through stabilization of GLI1 and modulation of EMT regulating proteins expression.

## Development of Pin1 Inhibitors for Hcc Treatments

Given the importance of PIN1 in HCC pathogenesis, PIN1 is an attractive drug target for HCC treatment. Our previous studies have shown that suppression of PIN1 by RNA interference in HCC cells reduces cell proliferation, inhibits colony formation in soft agar and enhances caspase-dependent apoptosis (Pang et al., 2006; Cheng et al., 2013). Moreover, PIN1 depletion results in a suppression of tumor growth and induction of tumor apoptosis in xenograft mouse model of HCC. Conceivably, targeting PIN1 is a potential therapeutic approach for HCC. Through screening of various chemical compound libraries, numerous PIN1 inhibitors have been identified to exert varying degrees of anti-proliferative effect on cancer cells by inhibiting PIN1 PPIase activity. PIN1 inhibitors can be subdivided into two groups (covalent and non-covalent) based on their binding to the PIN1 PPIase domain. After binding to the PIN1 PPIase domain, covalent PIN1 inhibitor (Juglone and KPT-6566) induces a covalent modification of thiol group of the cysteine residues in the PPIase domain (Hennig et al., 1998; Campaner et al., 2017). Through this structural modification of the catalytic domain, covalent PIN1 inhibitor irreversibly blocks the PIN1 PPIase domain and inhibits its activity. Most of the PIN1 inhibitors are non-covalent PIN1 inhibitors (e.g., PiB, ATRA, ATO, and API-1). Similar to covalent PIN1 inhibitor, non-covalent PIN1 inhibitor also directly binds to the PIN1 PPIase domain, but inhibits PIN1 activity in a competitive manner (Uchida et al., 2003; Wei et al., 2015; Kozono et al., 2018; Pu et al., 2018). However, there are limitations of these PIN1 inhibitors that restrict their clinical application (Table 3).

**TABLE 3:** Limitations of potential PIN1 inhibitors.

**Drug**	**Covalent or non-covalent**	**Details**	**Tested cancer types**	**Limitations**
Juglone	Covalent	Irreversible inhibits PIN1 PPIase activity Inhibits cell proliferation and xenograft tumor growth	HCC Prostate cancer	Non-specific inhibition of RNA polymerase II and Rab4
PiB	Non-covalent	Inhibits PIN1 PPIase activity and cell proliferation	Colon cancer	No testing in animal model
Dipentamethylene thiuram monosulfide (DTM)	Non-covalent	Inhibits PIN1 PPIase activity and cell proliferation	Colon cancer	No testing in animal model
TME-001	Non-covalent	Inhibits PIN1 PPIase activity and cell proliferation	Cervical cancer	No testing in animal model
5′-nitro-indirubinoxime (5′-NIO)	N.A.	Reduce PIN1 protein expression Inhibits cell proliferation	Lung cancer	No testing in animal model
KPT-6566	Covalent	Induce PIN1 protein degradation Inhibits cell proliferation More specific PIN1-inhibitory activity	Breast cancer Prostate cancer Lung cancer Pancreatic cancer	No testing in clinical trial

*HCC, Hepatocellular carcinoma; N.A., Not applicable.*

The first identified PIN1 inhibitor is juglone, which is a covalent PIN1 inhibitor exerts *in vitro* and *in vivo* anti-proliferative effect against various types of cancer cells by irreversibly inhibiting PIN1 PPIase activity (Hennig et al., 1998). Juglone not only inhibits cell proliferation in HCC and prostate cancer cells *in vitro*, but also suppresses tumor growth of prostate cancer in xenografting experiments (Lee et al., 2009; Kanaoka et al., 2015). Notwithstanding its potent anti-tumor effect, juglone is not suitable for clinical use due to the lack its specificity on PIN1 inhibition. Other than PIN1, Juglone has been found to inhibit the cellular functions of RNA polymerase II and Rab4 (Chao et al., 2001; Fila et al., 2008).

Several non-covalent PIN1 inhibitors such as PiB, dipentamethylene thiuram monosulfide (DTM) and TME-001 have been identified to inhibit PIN1 PPIase activity and suppress cancer cell proliferation. PiB and DTM are effective against cell proliferation in colon cancer while TME-001 is found to exert anti-proliferative effect on cervical cancer (Uchida et al., 2003; Tatara et al., 2009; Mori et al., 2011). In addition to directly inhibiting PPIase activity, 5′-nitro-indirubinoxime (5′-NIO) has been shown to reduce PIN1 protein expression, resulting in suppression of lung cancer cell proliferation (Yoon et al., 2012). A novel covalent PIN1 inhibitor, KPT-6566, has been found to induce PIN1 protein degradation, leading to inhibition of proliferation in cancer cells including breast, prostate, lung and pancreatic cancer (Campaner et al., 2017). Moreover, KPT-6566 exerts a higher anti-proliferative effect on PIN1-expressing cells than PIN1-silenced cells, suggesting that KPT-6566 has a more specific PIN1 inhibitory activity. Despite the promising anti-proliferative effects on cancer cells, the efficacy and safety of these inhibitors for cancer treatment in human remain to be verified in animal models and patients.

In addition to the development of more potent and specific PIN1 inhibitors, there were studies to demonstrate that several drugs, which are approved for other clinical indications, also possess inhibitory activity against PIN1. The potential values of these agents for HCC treatment are further discussed in the following sections (Table 4).

**TABLE 4:** Potential PIN1 inhibitors for HCC treatment.

**Drug**	**Covalent or non-covalent**	**Mechanisms of action**	**Clinical limitations**
Sorafenib	N.A.	FDA-approved for advanced HCC treatment Inhibits RAF/MEK/ERK and VEGF receptor tyrosine kinases Suppresses PIN1-mediated Mcl-1 protein stabilization Reduces PIN1 expression by Inhibiting Rb phosphorylation Enhances apoptosis and inhibits cell proliferation	Unsatisfactory response rate with only 12 weeks survival advantage (Llovet et al., 2008) Developing sorafenib resistance or enhancing metastatic traits (Chow et al., 2013)
All-trans retinoic acid (ATRA)	Non-covalent	Induces PIN1 protein degradation Inhibits cell proliferation, migration, invasion and metastasis of HCC cells Demonstrates an enhanced anti-cancer effect by encapsulated in a slow-releasing pellet and PLLA microparticle	Poor overall survival and unsatisfactory response rate (Meyskens et al., 1998)
Arsenic trioxide (ATO)	Non-covalent	Induces PIN1 protein degradation Inhibits HCC cancer cell proliferation and xenograft tumor growth Combined with ATRA to exert a synergistic effect in inhibiting HCC cell proliferation	Ineffective in a phase II clinical study (Lin et al., 2007)
API-1	Non-covalent	Restores PIN1-impaired microRNA biosynthesis by enhancing XPO5 pre-miRNA binding ability Inhibits HCC cancer cell proliferation and xenograft tumor growth Shows an enhanced anti-cancer activity by liposomal formulation (API-LP)	No testing in clinical trial

*N.A., Not applicable; FDA, US Food and Drug Administration; HCC, Hepatocellular carcinoma; Rb, Retinoblastoma; PLLA, Poly L-lactic acid; AQP9, Aquaporin 9; XPO5, Exportin-5.*

## Pin1 and Sorafenib

Sorafenib is a multi-tyrosine kinase inhibitor that is FDA-approved for the first-line treatment of advanced HCC. Through inhibition of the RAF/MEK/ERK and VEGF receptor tyrosine kinase signaling pathways, sorafenib has been shown to induce cell apoptosis, suppress cell proliferation, and inhibit tumor growth and angiogenesis in HCC cells (Liu et al., 2006). Given its inhibitory effect on ERK phosphorylation, sorafenib reduces the PIN1-induced stabilization of anti-apoptotic protein Mcl-1 by inhibiting the ERK-mediated phosphorylation of Mcl-1. This results in reduction of Mcl-1 protein expression, promotion of apoptosis, and inhibition of cell proliferation. Although a positive correlation between PIN1 and Mcl-1 has only been reported in human breast cancer, deregulated Mcl-1 expression is also commonly found in HCC (Fleischer et al., 2006; Sieghart et al., 2006). Theoretically, sorafenib may enhance apoptosis of HCC cells by impairing the interaction between PIN1 and Mcl-1. Thus, sorafenib may indirectly inhibit PIN1 function through targeting the phosphorylation of PIN1-interacting proteins.

In addition, sorafenib also reduces PIN1 mRNA and protein expression in HCC cells by inhibiting Rb phosphorylation (Zheng et al., 2017). As phosphorylated Rb releases E2F transcription factor and activates PIN1 expression, sorafenib may down-regulate PIN1 expression through targeting the Rb-E2F pathway. Furthermore, HCC cells with PIN1 depletion are more sensitive to sorafenib induced cell death, suggesting that some of the PIN1-interacting proteins associated with HCC pathogenesis may not be the targets of sorafenib. Therefore, it is speculated that PIN1 inhibitor together with sorafenib may have synergistic therapeutic effects against HCC. A recent study by Zheng et al. (2017) has demonstrated that combined treatment of sorafenib with a PIN1 inhibitor, all-*trans* retinoic acid (ATRA) [details will be discussed below], exerts a synergistic effect in inhibiting cell proliferation and xenograft tumor growth in HCC as compared with sorafenib or ATRA alone. Moreover, combination of sorafenib and ATRA results in a synergistic inhibition of PIN1 protein expression and various PIN1-mediated oncogenic pathways. In a clinical study, although HCC patients receiving sorafenib monotherapy have longer overall survival, the response rate remains unsatisfactory with only 12 weeks survival advantage (Llovet et al., 2008). Sorafenib monotherapy also involves in development of sorafenib resistance or in enhancing the metastatic traits of HCC cells (Chow et al., 2013; Liang et al., 2013). Thus, the study of combination therapy with sorafenib and various PIN1 inhibitors may improve the overall survival in HCC patients and minimize the risk of drug resistance and metastasis.

## Pin1 and Atra

ATRA is first identified as a therapeutic agent for acute promyelocytic leukemia (APL). It inhibits cell proliferation of APL cells by inducing terminal differentiation of APL cells. A recent study by Wei et al. (2015) has demonstrated a PIN1-inhibitory function of ATRA against cancer cells. ATRA directly binds to the PIN1 PPIase domain, causing PIN1 isomerase inhibition and PIN1 protein degradation. ATRA-induced PIN1 degradation results in inhibition of multiple cancer-driving pathways and suppression of proliferation in APL cells *in vitro* and *in vivo*. For HCC cells, ATRA has been demonstrated to exert a profound inhibitory effect on cell proliferation *in vitro* (Cui et al., 2016). In addition, HCC cells with PIN1 depletion are more resistant to the cytotoxic effect of ATRA than control cells, suggesting that ATRA-induced PIN1 degradation plays a critical role in the suppression of HCC cell proliferation (Yang et al., 2018). Moreover, ATRA does not show any growth-inhibitory effect on normal liver cells, further demonstrating its specificity toward HCC cancer cells. Importantly, ATRA also exhibits an inhibitory effect on migration, invasion, and lung metastasis of HCC cells by inducing protein degradation of PIN1 (Wang et al., 2019).

However, previous clinical study has demonstrated that ATRA was ineffective in patients with HCC, as evidenced by demonstration of a poor overall survival and unsatisfactory response rate (Meyskens et al., 1998). The efficacy of ATRA in HCC treatment is limited because of its short half-life of 45 min in humans and its rapid metabolism by liver. Therefore, it is necessary to develop a more stable ATRA formulation for clinical application. A slow-releasing ATRA formulation has been developed by encapsulating ATRA in the acid form of vitamin A pellets. In contrast to free ATRA, the slow-releasing ATRA pellet is more stable in animals and can maintain ATRA plasma concentration in a steady level (Liao et al., 2017). Therefore, a minimum effective dose of the slow-releasing ATRA formulation can be applied to minimize its toxic effects to animals. More importantly, the slow-releasing ATRA formulation has been found not only to induce PIN1 degradation, but also reduce tumorigenicity in xenograft mouse model of HCC. A more recent study has identified a novel controlled release formulation of ATRA that exerts a more potent anti-proliferative effect against HCC cells (Yang et al., 2018). This novel formulation is processed through encapsulation of ATRA into the poly L-lactic acid (PLLA) microparticles. In comparison with slow-releasing ATRA formulation, the treatment of ATRA-PLLA microparticles shows a more significant inhibition of xenograft tumor growth and reduction of PIN1 protein expression. Moreover, injection of ATRA-PLLA microparticles into mice achieves a higher ATRA plasma concentration in a steady level as compared with implantation of slow releasing ATRA pellet. Notably, ATRA-PLLA microparticle showing more potent anti-cancer efficacy is encapsulated with a lower concentration of ATRA (2 mg) while slow releasing ATRA pellet has a higher ATRA concentration (5 mg). Thus, these findings demonstrate that ATRA is an attractive PIN1-targeting therapeutic drugs against HCC and the development of stable encapsulated ATRA is a promising strategy to improve the efficacy and safety of its use.

## Pin1 and Ato

Arsenic trioxide (ATO), is a FDA approved drug used for the treatment of APL that is refractory to or relapsed after ATRA therapy. The anti-cancer effect of ATO mainly depends on its ability to induce ubiquitin-dependent proteasomal degradation of various oncogenic proteins, including promyelocytic leukemia-retinoic acid receptor-alpha (PML-RARA) in APL, cyclin D1 in mantle cell lymphoma, and nucleophosmin-anaplastic lymphoma kinase (NPM-ALK) in anaplastic large cell lymphoma (Shao et al., 1998; Lo and Kwong, 2014; Piao et al., 2017). Through the proteasome pathway, ATO has also been found to induce PIN1 degradation by directly binding to the PIN1 PPIase domain (Kozono et al., 2018). ATO-induced PIN1 degradation results in the suppression of multiple PIN1-mediated oncogenic pathways and inhibition of breast cancer cell proliferation *in vitro* and *in vivo*. For HCC cells, ATO inhibits cell proliferation and xenograft tumor growth through triggering caspase-dependent apoptosis (Kito et al., 2002, 2003; Sadaf et al., 2018). However, a phase II clinical study has showed that ATO monotherapy is ineffective as a treatment for HCC (Lin et al., 2007).

The cytotoxic effect of ATO partly depends on its cellular uptake that is mediated by a transmembrane arsenic transporter, aquaporin 9 (AQP9) (Leung et al., 2007). The ATO-induced cell death is highly associated with expression level of AQP9, and AQP9 expression level varies between different types of cancer cells. Up-regulation of AQP9 expression may enhance the cytotoxic effect of ATO against cancer cells. In addition to its PIN1-inhibitory activity, ATRA has been shown to increase AQP9 expression to promote ATO uptake into the cells. As a result of enhancing cellular uptake of ATO, combined treatment of ATO with ATRA synergistically reduces PIN1 expression, suppresses multiple PIN1-regulated oncogenic pathways, and inhibits breast cancer cell proliferation *in vitro* and *in vivo* as compared with ATO or ATRA alone (Kozono et al., 2018). Although a clinical study for ATO-ATRA combination therapy against HCC has yet to be conducted, experiments have demonstrated that this combined treatment exerts a synergistic effect in inhibition of cell proliferation and promotion of apoptosis in HCC cells *in vitro* (Lin et al., 2005; Wei et al., 2014).

## Pin1 and API-1

Most of the identified PIN1 inhibitors exert their anti-proliferative effect against cancer cells in a PIN1-dependent manner with a higher inhibition of cell proliferation in PIN1-expressing cells than PIN1-depleted cells. A recent study has discovered a novel PIN1 inhibitor, API-1, with a potent anti-proliferative effect in HCC cells, and its anti-proliferative activity is dependent on both PIN1 expression and XPO5 phosphorylation (Pu et al., 2018). HCC cells with higher PIN1 expression and enhanced XPO5 phosphorylation are more sensitive to API-1 treatment than those with low PIN1 expression and/or reduced XPO5 phosphorylation. As previously described, XPO5 is responsible for the nuclear-cytoplasmic export of pre-miRNA to facilitate miRNA maturation. PIN1 interacts with phosphorylated XPO5 to impair its pre-miRNA binding ability, resulting in reduction of pre-miRNA export and miRNA expression. Due to its PIN1-inhibitory activity, API-1 has been found to increase XPO5-mediated pre-miRNA export from the nucleus to the cytoplasm and restore biosynthesis of tumor suppressive miRNAs in HCC cells. Consequently, treatment of API-1 in HCC cells results in reduction of cell proliferation and suppression of xenograft tumor growth through restoration of PIN1-impaired miRNA biosynthesis.

In addition, liposomal formulation of API-1 further enhances its anti- HCC effects *in vitro* and *in vivo* (Sun et al., 2019). Encapsulation of API-1 by liposome (API-LP) results in an increase of API-1 solubility and a slower rate of API-1 drug release, leading to an improved bioavailability of API-LP in animals. Similar to unencapsulated API-1, API-LP restores XPO5-mediated pre-miRNA nuclear-cytoplasmic export and miRNA biogenesis through the inhibition of PIN1 activity. Due to its improved bioavailability, API-LP shows a more significant suppression of tumor growth of HCC xenografts as compared with unencapsulated API-1. More importantly, API-LP shows no apparent toxicity to the mice as it does not cause any necrotic damage to the tissues in mouse major organs such as heart, liver, spleen, lung and kidney. Although further study is required to evaluate the clinical efficacy and safety of API-LP for HCC patients, the liposomal formulation provides new insights into the development of a potent PIN1 inhibitor with enhanced bioavailability against HCC in animals and humans.

## Conclusion and Future Perspectives

Given the oncogenic role of PIN1 in promoting hepatocarcinogenesis, targeting PIN1 is a potential therapeutic approach against HCC. Through high-throughput screening technology, it is possible to identify novel and potent PIN1 inhibitors from different chemical compound libraries. However, the clinical use of PIN1 inhibitor is not only determined by its anti-cancer activity, but also depends on its *in vivo* bioavailability. Poor aqueous solubility and chemical instability of various identified PIN1 inhibitors limit their clinical applications. The development of microparticle (ATRA-PLLA) or liposome (API-LP) encapsulated PIN1 inhibitors show great superiority in the inhibition of HCC xenograft tumor growth by improving the bioavailability of PIN1 inhibitors in animals. Further study and work are required to develop various encapsulation methods for enhancing the anti-cancer effect of PIN1 inhibitors.

In recent years, a number of new molecular targeting drugs have been approved to treat patients with advanced-stage HCC (Table 1). Regorafenib is one of these new drugs that has been recommended as a second-line treatment option for sorafenib-resistant unresectable HCC. In a randomized clinical trial, regorafenib has been shown to improve overall survival in HCC patients that progressed following first-line sorafenib treatment (Bruix et al., 2017). However, similar to sorafenib treatment, regorafenib resistance will develop with time in HCC patients. A recent study has demonstrated that the development of regorafenib-resistant HCC cells by continuous low-dose treatment of regorafenib results in up-regulation of PIN1 (Wang et al., 2019). Although the relationship between PIN1 expression and acquired regorafenib-resistance in HCC remains to be further investigated and confirmed, the role of PIN1 in drug resistance in HCC would be an interesting topic that is worth exploring. A better understanding of the molecular mechanisms of PIN1 that lead to acquired drug resistance is critical to support the potential use of various PIN1 inhibitors as a second-line treatment option for drug-resistant HCC.

## Author Contributions

C-WC and ET wrote and approved the review.

## Conflict of Interest

The authors declare that the research was conducted in the absence of any commercial or financial relationships that could be construed as a potential conflict of interest.
